# A conserved motif within *cox 2* allows broad detection of economically important fruit flies (Diptera: Tephritidae)

**DOI:** 10.1038/s41598-018-20555-2

**Published:** 2018-02-01

**Authors:** Fan Jiang, Liang Liang, Zhihong Li, Yanxue Yu, Jun Wang, Yuping Wu, Shuifang Zhu

**Affiliations:** 1 Chinese Academy of Inspection and Quarantine, Beijing, 100176 China; 2 Key Laboratory of Agro-Products Postharvest Handling, Ministry of Agriculture, Chinese Academy of Agricultural Engineering, Beijing, 100121 China; 3 College of Plant Protection, China Agricultural University, Beijing, 100193 China

## Abstract

The genera *Anastrepha*, *Bactrocera*, *Ceratitis*, *Dacus* and *Rhagoletis* in the family Tephritidae order Diptera are economically important, worldwide distributed and cause damage to a large number of commercially produced fruits and vegetables. China had regulated these five genera as quarantine pests, including the species *Carpomya vesuviana*. An accurate molecular method not depending on morphology able to detect all the quarantine fruit flies simultaneously is required for quarantine monitoring. This study contributes a comparative analysis of 146 mitochondrial genomes of Diptera species and found variable sites at the mt DNA *cox2* gene only conserved in economically important fruit flies species. Degenerate primers (TephFdeg/TephR) were designed specific for the economically important fruit flies. A 603 bp fragment was amplified after testing each of the 40 selected representative species belonging to each economically important Tephritid genera, no diagnostic fragments were detected/amplified in any of the other Tephritidae and Diptera species examined. PCR sensitivity assays demonstrated the limit of detection of targeted DNA was 0.1 ng/μl. This work contributes an innovative approach for detecting all reported economically important fruit flies in a single-step PCR specific for reported fruit fly species of quarantine concern in China.

## Introduction

The family Tephritidae, the true fruit flies, is among the largest families in Diptera. Tephritidae includes over 5000 species classified in 500 genera^1,2^, is distributed worldwide except Antarctica^1^. Most species in the true fruit flies group cause significant damage to fruit and vegetable production and many species have spread to new regions where they have become a significant threat to global horticulture^1,3^. Relevant pest genera are *Anastrepha* Schiner, *Bactrocera* Macquart, *Ceratitis* Macleay, *Dacus* Fabricius and *Rhagoletis* Loew which are considered economically important^1^. China had regulated these five genera as quarantine pest, and had included other species from other genera like *Carpomya vesuviana* Costa. *Bactrocera dorsalis* (Hendel), *B. cucurbitae* (Coquillett) and *Carpomya vesuviana*, which are considered invasive species and are managed carefully by the Ministry of Agriculture of China. The movements of these devastating fruit flies have increased in recent years due to international trade and globalization. Rigorous quarantine measures are in place in many countries to limit the international movement of these insects across countries and/or continents. Several International Standards for Phytosanitary Measures (ISPM) have been created by the International Plant Protection Convention (IPPC) to address the problem of invasive fruit flies. These international standards involve aspects related to pest risk managements, quarantine treatment, monitoring and control.

Rapid detection of economically important fruit flies is a pivotal measure to prevent the introduction of potentially invasive species. Indeed, this is the first and most effective approach to minimize the harm caused by invasive fruit flies^4^. Molecular detection methods are effective procedures to identify fruit flies rapidly overcoming life-stage restrictions because are based on species specific nucleotide sequence data. Moreover, there is no need to rear the intercepted fruit fly eggs, larvae or pupae to adults, which is a time-consuming and sometimes unsuccessful process^5^.

Current molecular methods for fruit flies identification focus on DNA barcoding, PCR or quantitative PCR with species-specific primers and probes. DNA barcoding uses mitochondrial (mt) DNA and focuses on the *cox1* gene, which allows discrimination of most fruit flies, except several species complex which are difficult to identify in to species level^6,7^. However, the carry-on time for sequencing can take several hours to days causing delay downstream quarantine procedures and decision making. Universal barcode primers^8^ for metazoan invertebrates did not consistently discriminate some *Ceratitis* species such as *C. cristata*^6^. To bypass the need for post sequencing, end point PCR or quantitative PCR processing, species-specific markers were developed for one or several specific species only^9–15^. Jiang *et al*. developed a standardized reaction system for simultaneous detection of 27 fruit fly species intercepted at ports in China based on a microfluidic dynamic array of mt DNA *cox1* gene that uses species-specific primers and probes for quarantine concern^16^. However, these molecular methods are yet insufficient to detect all economically important fruit flies at the border during quarantine inspections.

Focus toward mitochondrial genes have been consistently matter of attention for fruit flies molecular identification studies^2,6,7,17,18^. Most of these studies had targeted the cytochrome *c* oxidase subunit 1 (*cox1*) as a near-exclusive data source for fruit fly species identification and species delimitation since DNA barcoding was proposed^2,6,7,17–20^. However, for higher taxons, such as Tephritidae, there is no suitable markers that can be used for simultaneous broad detection of economically important fruit flies^21,22^. Exploring mitochondrial genomes provide more information about genetic variability than shorter sequences of individual genes such as *cox1* and are widely used for molecular systematics at different taxonomic levels^23^. We hypothesize that analyzing the whole mitochondrial genomes of Tephritidae unfolded toward some Diptera will provide new markers of value for broad detection of economically important fruit flies.

In this study, mitochondrial genomes of Diptera sourced in GenBank (https://www.ncbi.nlm.nih.gov/genbank/) were aligned aiming for variable landmarks in Diptera but conserved in economically important fruit flies to develop a ‘specific broad detection’ pair of primers for simultaneous broad detection of all the economically important fruit flies in a single-step end point PCR.

## Results

### DNA extraction and quality

DNA was extracted from legs of adults of both sex and the obtained DNA concentration varied from 70–120 ng/μl. The DNA quality was determined at A260/A280 and ranged between 1.7–2.0.

### Sequence alignment and primer design

The complete mitochondrial genomes of 146 Diptera species were sourced from GenBank and the pool included 16 economically important Tephritidae species: *Bactrocera (Bactrocera) arceae* (Hardy & Adachi)^24^, *B. (B.) carambolae* Drew & Hancock, *B. (B.) correcta* (Bezzi), *B. (B.) dorsalis*^25^, *B. (B.) latifrons* (Hendel)^26^, *B. (B.) melastomatos* Drew & Hancock^26^, *B. (B.) tryoni* (Froggatt)^27^, *B. (B.) umbrosa* (Fabricius)^26^, *B. (B.) zonata* (Saunders)^28^, *B. (Daculus) oleae* (Gmelin)^27,29^, *B. (Tetradacus) minax* (Enderlein)^30^, *B. (Z.) cucurbitae*^31^, *B. (Z.) diaphora* (Hendel)^32^, *B. (Z.) scutellata* (Hendel), *B. (Z.) tau* (Walker)^33^ and *Ceratitis (Ceratitis) capitata* (Wiedemann)^34^. Software assisted and visual analysis of the Diptera mitochondrial genomes allowed the detection of polymorphic sites. The sequence alignment is provided in Appendix S1. To specifically amplify all economically important Tephritidae species, degenerate primers were designed to target a DNA fragment within the mt DNA *cox2* gene. The location of the degenerate primer pair is provided in Appendix S1. The sense primer spans from loci 5331 to 5390. The antisense primer spans from loci 5981 to 6003. Location refers to accession DQ 845759, *Bactrocera dorsalis*. This broad detection degenerate primer pair was labeled TephFdeg (sense) and TephR (antisense). Detailed information about this primer pair is provided in Table 1.

**Table 1:** Sequence, localization and thermodynamic parameters of primers used.

Primer	Sequence 5′-3′	Location in DQ845759^*^	Fragment Size (bp)	Tm (°C)	GC%	3′ΔG (kcal/mol)
TephFdeg	GACAACATGAGCHGSHYTHGGBCT	3142–3165	603	55.1 to 57.6	41.7	−7.0
TephR	GCTCCACAAATTTCTGAACATTG	3722–3744		56.6	39.1	−5.9

^*^DQ845759 complete mitochondrial genome of *Bactrocera dorsalis*.

### Degenerate primers specificity

**Figure 1:**
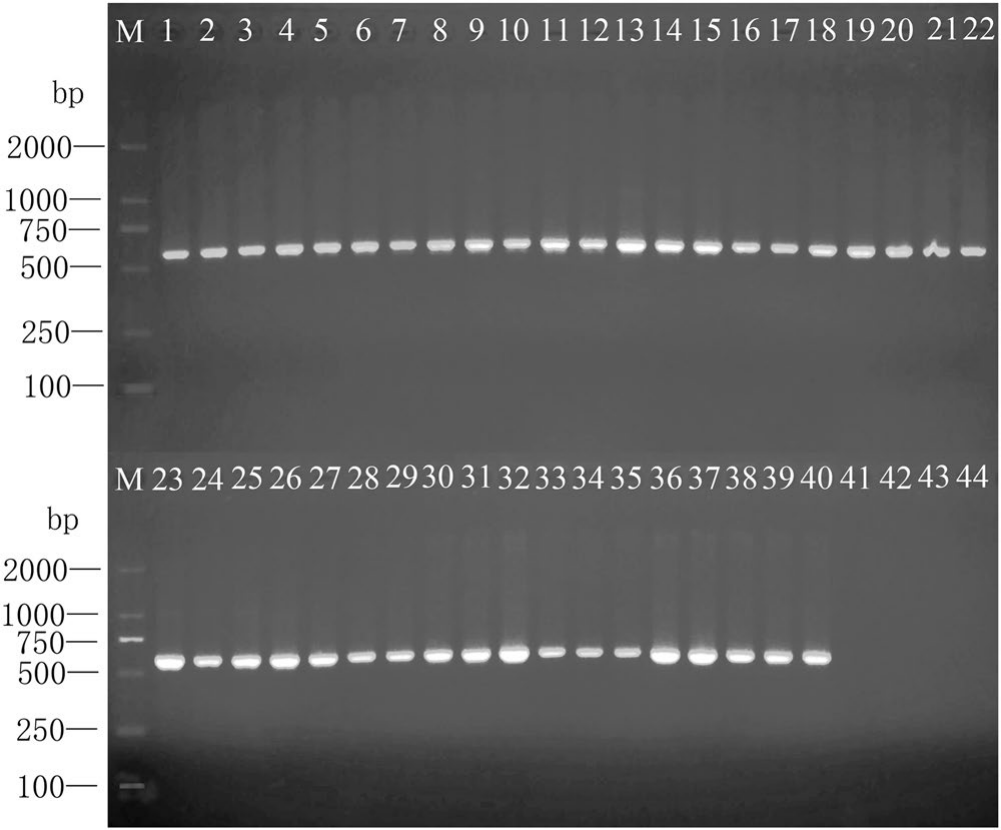
Specificity of primers TephFdeg/TephR for fruit fly broad detection. Lanes 1 to 43 correspond to species No. 1 to 43 listed in Table 2; Lane 41 is *P. utilis*, Lane 42 is *A. striates* and Land 43 is *D. melanogaster*, three non economically important fruit flies; Lane 44 is ddH_2_O non template control (NTC); and Lane M is a D2000 DNA ladder.

Specificity test showed the amplification of a single 603 bp DNA product in each of the economically important Tephritidae species tested. No amplification was detected in of the non-economic Tephritidae and Diptera species tested (Fig. 1). These results were consistent after three replications. BLAST alignments of all obtained fragment sequences amplified by primer set TephFdeg/TephR matched with mitochondrial genes of Tephritidae species with 89%–100% identity and 0.0 E value. Detailed information of the compared species, including accession number, score, query cover, E value and identity is listed in the Supplementary Table S1.

### Primers sensitivity

**Figure 2:**
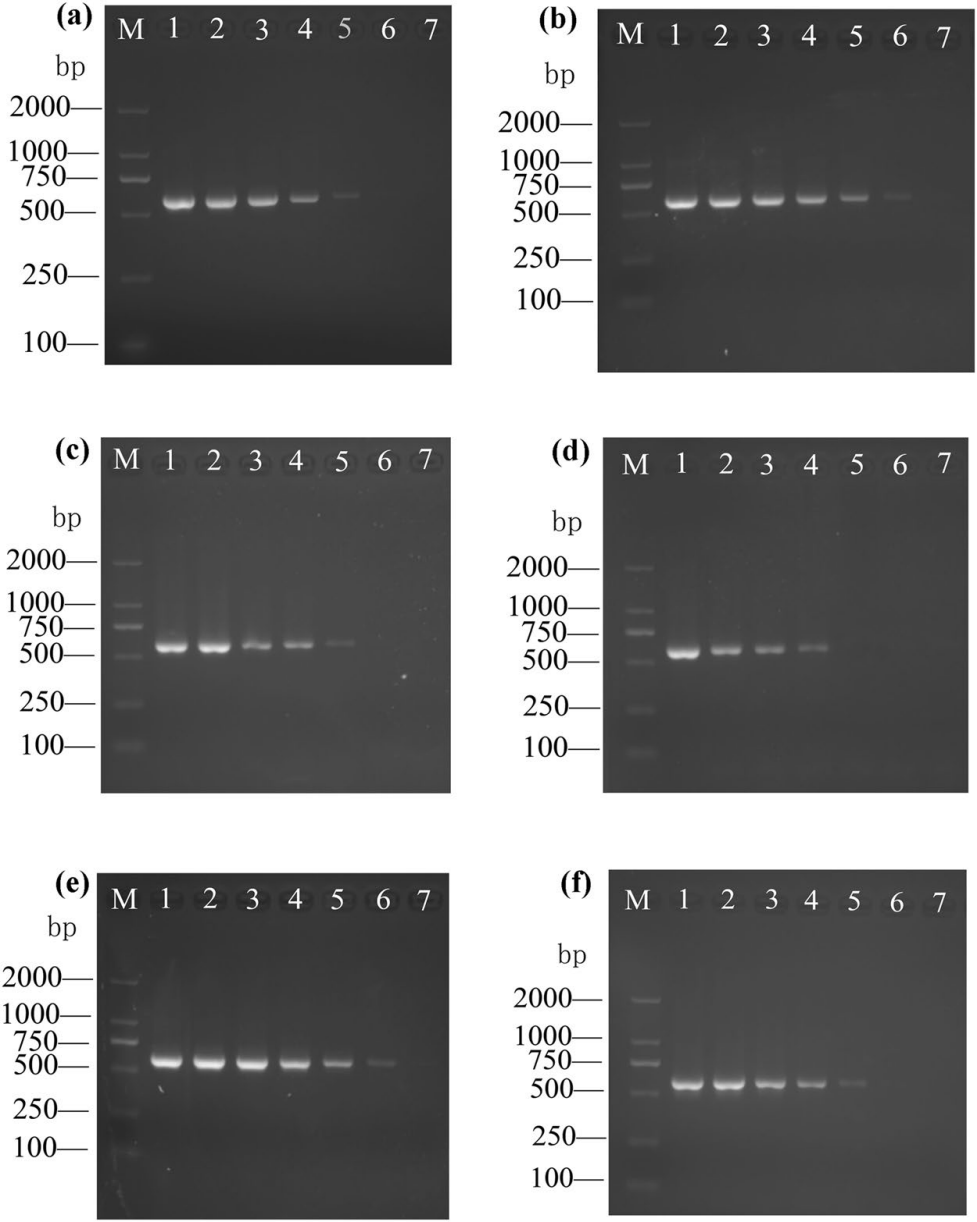
Sensitivity of primers TephFdeg/TephR. Template DNA are: (**a**) *Anastrepha* spp., (**b**) *Bactrocera* spp., (**c**) *Carpomya* spp., (**d**) *Ceratitis* spp., (**e**) *Dacus* spp. and (**f**) *Rhagoletis* spp.; DNA serial dilutions are as follows: Lane 1: 100 ng/μl, Lane 2: 10 ng/μl, Lane 3: 1 ng/μl, Lane 4: 0.1 ng/μl, Lane 5: 0.01 ng/μl, Lane 6: 0.001 ng/μl, Lane 7: ddH_2_O NTC, Lane M: is a D2000 DNA ladder.

Three sensitivity assays were performed and showed the limit of detection using *Bactrocera* spp. and *Dacus* spp. as reference species was 0.001 ng/μl, *Anastrepha* spp., *Carpomya* spp. and *Rhagoletis* spp. was 0.01 ng/μl, and *Ceratitis* spp. was 0.1 ng/μl (Fig. 2, one species of each genus as an example, the results of all tested species were shown in Supplementary Figure S1–S40). Overall, the limit of detection of all economically important Tephritidae species is 0.1 ng/μl.

## Discussion

A pair of degenerate primers was designed and a single-step PCR method was developed to detect broad range of economically important fruit flies simultaneously. The method amplifies a single product of 603 bp. The method has application monitoring quarantine samples in China and elsewhere. It is a simple method transferable to non-taxonomists and particularly useful for detection of important fruit flies and their immature stages or partially damaged specimens missing characteristics diagnostic body parts which cannot be identified by traditional means. Previously reported molecular methods developed for identification of Tephritidae species detected several species only. This new proposed method is simple and requires no further sequencing of the amplified products, and requires only widely used PCR equipment, which facilitates the process of detection in any applied quarantine laboratory.

To demonstrate the accurate multi-target detection of fruit flies, the specificity and broad detection of the primers is the most important element. During the process of the primer design, 146 Diptera mitochondrial genomes were compared to ensure the specificity and broad detection of designed primers. However, newly released mitochondrial genomes of *Anastrepha fraterculus* (Wiedemann)^35^, *B. (B.) invadens* Drew, Tsuruta & White^36^, *B. (Paradacus) depressa* (Shiraki)^37^, *B. (Z.) caudata* (Fabricius)^38^ and *Dacus (Callantra) longicornis* Wiedemann^39^ were not included since were not available, nonetheless, TephFdeg/TephR primers amplify *A. fraterculus*, *B. caudata* and *D. longicornis* during the specificity test (Specimens 1, 9 and 38, Fig. 1). The competence of primers TephFdeg/TephR to amplify target fragments from *B. depressa* and *B. invadens* was verified in silico according to the sequence alignments analysis. This PCR detection method was validated with 40 economically important fruit flies. Only the expected product size was amplified and their identity was confirmed by sequencing. The NCBI accession numbers of all detection/ diagnostic fragments are listed in Table 2. Primers TephFdeg/TephR did not amplify three different species of non economic fruit flies as predicted. Primers TephFdeg/TephR showed to be reliable, since all specificity assays were successfully replicated three times.

**Table 2:** Specimens tested with primers TephFdeg/TephR.

Specimen No.	Family	Genus	Species	Stage	Source/country	Accession numbers
1	Tephritidae	Anastrepha*	*A. fraterculus*	Adult	Intercepted	MG020783
2			*A. obliqua*	Larva	Intercepted	MG020784
3			*A. sp1*	Adult	Intercepted	MG020785
4			*A. sp2*	Larva	Intercepted	MG020786
5		Bactrocera*	*B. albistrigata*	Adult	Indonesia	MG020787
6			*B. atrifacies*	Adult	China	MG020788
7			*B. bezziana*	Adult	China	MG020789
8			*B. carambolae*	Adult	Surinam	MG020790
9			*B. caudata*	Adult	Thailand	MG020791
10			*B. cilifera*	Adult	China	MG020792
11			*B. correcta*	Adult	China	MG020793
12			*B. cucurbitae*	Adult	China	MG020794
13			*B. diversa*	Adult	China	MG020795
14			*B. dorsalis*	Adult	China	MG020796
15			*B. hochii*	Adult	China	MG020797
16			*B. kandiensis*	Adult	Sri Lanka	MG020798
17			*B. latifrons*	Adult	Malaysia	MG020799
18			*B. minax*	Adult	China	MG020800
19			*B. oleae*	Adult	Italy	MG020801
20			*B. rubigina*	Adult	China	MG020802
21			*B. scutellaris*	Adult	China	MG020803
22			*B. scutellata*	Adult	China	MG020804
23			*B. synnephes*	Adult	China	MG020805
24			*B. tau*	Adult	China	MG020806
25			*B. thailandica*	Adult	Thailand	MG020807
26			*B. tryoni*	Adult	Australia	MG020808
27			*B. tsuneonis*	Adult	China	MG020809
28			*B. umbrosa*	Adult	Thailand	MG020810
29			*B. wuzhishana*	Adult	China	MG020811
30			*B. yoshimotoi*	Adult	China	MG020812
31			*B. zonata*	Adult	Pakistan	MG020813
32		Carpomya*	*C. vesuviana*	Adult	China	MG020814
33		Ceratitis*	*C. capitata*	Adult	Intercepted	MG020815
34			*C. cosyra*	Larva	Intercepted	MG020816
35			*C. rosa*	Larva	Intercepted	MG020817
36		Dacus*	*D. bivittatus*	Larva	Intercepted	MG020818
37			*D. ciliatus*	Larva	Intercepted	MG020819
38			*D. longicornis*	Adult	China	MG020820
39		Rhagoletis*	*R. pomonella*	Larva	Intercepted	MG020821
40			*R. sp*.	Larva	Intercepted	MG020822
41		*Procecidochares*	*P. utilis*	Adult	China	
42	Pyrgotidae	*Adapsilia*	*A. striatis*	Adult	China	
43	Drosophilidae	*Drosophila*	*D. melanogaster*	Adult	China	

*Economically important fruit flies tested in this study.

The sensitivity of primers TephFdeg/TephR is 0.1 ng/μl of template DNA and was determined for 40 species of six economically important fruit fly genera (Fig. 2). Highly sensitive detection is very important when testing a trace amount of DNA template. A previous study showed at least 5 ng/μl DNA are obtained from any life stages of a single fruit fly sample^12^. DNA extraction would take time and energy, for quarantine application, quick methods for DNA extraction will also be needed. The new methodology developed here can detect the target species as long as the concentrate of DNA is above 0.1 ng/μl whatever DNA extraction method used. We believe any quick DNA extraction methods should obtain DNA from a single fruit fly with the concentrate higher than 0.1 ng/μl. Therefore, the molecular method described in this study is able to detect the presence of economically important fruit flies in any developmental stages even from a single egg.

This fruit fly detection method was developed particularly for quarantine purposes. The genera *Anastrepha*, *Bactrocera*, *Ceratitis*, *Dacus* and *Rhagoletis* are considered quarantine pests in China and other countries and detection at genus level is sufficient. If compared with the species-specific primers, the TephFdeg/TephR primers in this study are relatively universal and can amplify various species of quarantine fruit flies in China. Primers TephFdeg/TephR are more specific than the universal barcode primers Lco1490/Hco2198 which are widely used to amplify invertebrates^8^. This study did not focus on the species confirmation. For the further species confirmation, the species-specific primers and/or probes can be used to identify specific species. DNA barcoding can also be used for most Tephritidae species. However, DNA barcoding based on mt DNA *cox1* gene may not allow a sharp discrimination of economically important Tephritidae species, because the existence of species complex and the lack of barcoding primers for some species^6,7^. The primers designed in this study overcome the described shortcomings and may provide a potential marker within mt DNA *cox2* gene that facilitates the detection of economically important fruit flies at ports of entry. A more comprehensive analysis including tree-based, distance-based and character-based methods will be required to verify further uses of this new *cox2* marker regarding *cox1*. However, a special database with obtained markers from all Tephritidae would contribute an important fruit flies identification resource particularly for quarantine purpose. Meanwhile, a standardized quarantine and identification procedure for all quarantine fruit flies can be implemented to provide a decision support tool for global import and export trade.

## Materials and Methods

### Sample collection

Thirty-two species were obtained from field collections, and the remaining 11 species were intercepted at ports of entry in China. The origin and stage information of the samples tested in this study is listed in Table 2. The larvae intercepted at ports of entry were reared to adult stage for identification. All adult samples were preserved in 100% ethanol and stored at −20 °C at the Chinese Academy of Inspection and Quarantine until use. The adult specimens were identified according to available taxonomic keys before initiating the molecular works^1,40^.

### Broad detection primer design

The mitochondrial genomes of Diptera were downloaded from GenBank. The sequence alignment of Diptera mitochondrial genomes was performed using DNAMAN (Demonstration Version)^41^. Sites with variability in Diptera, but showing conservation in economically important fruit flies only were visually identified. The broad detection primers were visually designed without software assistance during the analysis of the aligned sequences covering these sites of interest. Primer thermodynamics, GC contents and the false priming efficiency of the broad detection primer sequences were checked with OLIGO 7.0^42^. Primers were synthesized by Sangon Biotech (Shanghai) Co., Ltd.

### Broad detection, specificity and sensitivity of the primer test

Total genomic DNA was extracted from legs of individual adults using the TIANamp Genomic DNA kit (TIANGEN, China). DNA concentrations were estimated by spectrophotometry (NanoDrop 1000 spectrophotometer, Thermo Scientific, USA). The rest of the insect body and DNA were kept at −20 °C as voucher.

The specificity of the broad detection primers was tested performing PCR with the samples listed in Table 2. The criterion for primer selection was broad detection of only economically important Tephritidae samples, and no amplification of other Tephritidae and Diptera species (e.g., *Procecidochares* spp., *Adapsilia* spp. and *Drosophila* spp.). The PCR reaction was performed in a total volume of 25 μl, including 12.5 μl Taq PCR Master Mix (TIANGEN, China), 0.5 μl of each primer (10 μM), 1 μl of template DNA, and 10.5 μl of distilled water. PCR cycling conditions consisted of an initial denaturation at 95 °C for 3 min, followed by 35 cycles of denaturation at 95 °C for 15 s, annealing and extension at 60 °C for 1 min, and a final extension at 60 °C for 1 min. To confirm products were generated by the broad detection primers, each amplified product was sequenced using the same PCR primers. Sequencing services were by Sangon Biotech (Shanghai) Co., Ltd, and the derived sequences were submitted to GenBank for accession numbers (Table 2) and compared with known sequences in the NCBI database. The specificity test was repeated three times and three individuals of each population were used to ensure the accuracy and reliability of the result.

To determine the sensitivity of the selected multi-target primers with the above-mentioned PCR condition, a dilution series of template DNA were dissolved in TE buffer from one of each species used. The DNA concentrations were 100 ng/μl, 10 ng/μl, 1 ng/μl, 0.1 ng/μl, 0.01 ng/μl, 0.001 ng/μl, respectively. Double distilled water was used as a negative control. The sensitivity test was also repeated three times for each species to make sure that the result was accurate and reliable.

### Data Availability

The datasets generated during and analyzed during the current study are available from the corresponding author on reasonable request.

## Electronic supplementary material

Supplementary Information
