# Supplementary material for: A conserved motif within cox 2 allows broad detection of economically important fruit flies (Diptera: Tephritidae)
Source: Sci Rep. 2018 Feb 1;8:2077. doi: 10.1038/s41598-018-20555-2 (PMC5794786; doi:10.1038/s41598-018-20555-2)
Supplement: Supplementary file 1 — Supplementary Information [file 41598_2018_20555_MOESM1_ESM.pdf]

A conserved motif within *cox 2* allows broad detection of economically important fruit flies (Diptera: Tephritidae)

Fan Jiang[1†], Liang Liang[2†], Zhihong Li[3*], Yanxue Yu[1], Jun Wang[1], Yuping Wu[1*], Shuifang Zhu[1*]

[1]Chinese Academy of Inspection and Quarantine, Beijing 100176, China.

[2]Key Laboratory of Agro-Products Postharvest Handling, Ministry of Agriculture, Chinese Academy of Agricultural Engineering, Beijing 100121, China.

[3]College of Plant Protection, China Agricultural University, Beijing 100193, China.

[*] Corresponding author: zhusf@caiq.gov.cn; wuyuping@caiqtest.com; lizh@cau.edu.cn
[†] These authors contributed equally to this work.

Table S1. BLAST alignment results of all positive fragment sequences generated by primer set TephFdeg/TephR

No.	Genus	Tested species	Stage	Source	Compared species	Max score	Total score	Query cover	E value	Ident	Accession
1	*Anastrepha*	*A. fraterculus*	Adult	Intercepted	*A. fraterculus*	955	955	100%	0.0	98%	KX926433
2		*A. obliqua*	Larva	Intercepted	*A. fraterculus*	955	955	100%	0.0	98%	KX926433
3		*A.* sp1	Adult	Intercepted	*A. ludens*	876	876	86%	0.0	99%	AB192462
4		*A.* sp2	Larva	Intercepted	*A. fraterculus*	721	721	98%	0.0	90%	KX926433
5	*Bactrocera*	*B. albistrigata*	Adult	Indonesia	*B. frauenfeldi*	966	966	100%	0.0	98%	JQ671171
6		*B. atrifacies*	Adult	China	*B. caudata*	861	861	100%	0.0	95%	KT625492
7		*B. bezziana*	Adult	China	*B. depressa*	1011	1011	100%	0.0	99%	AB092483
8		*B. carambolae*	Adult	Surinam	*B. carambolae*	1011	1011	100%	0.0	99%	EF014414
9		*B. caudata*	Adult	Thailand	*B. caudata*	1027	1027	100%	0.0	100%	KT625492
10		*B. cilifera*	Adult	China	*B. diaphora*	712	712	100%	0.0	90%	KT159730
11		*B. correcta*	Adult	China	*B. correcta*	1011	1011	100%	0.0	99%	JX456552

12	*B. cucurbitae*	Adult	China	*B. cucurbitae*	1027	1027	100%	0.0	100%	HQ664485
13	*B. diversa*	Adult	China	*B. diversa*	1005	1005	100%	0.0	99%	KJ833875
14	*B. dorsalis*	Adult	China	*B. dorsalis*	1022	1022	100%	0.0	99%	AB090272
15	*B. hochii*	Adult	China	*B. tau*	743	743	99%	0.0	91%	KP711431
16	*B. kandiensis*	Adult	Sri Lanka	*B. dorsalis*	1011	1011	100%	0.0	99%	AB090272
17	*B. latifrons*	Adult	Malaysia	*B. latifrons*	1011	1011	100%	0.0	99%	KT881556
18	*B. minax*	Adult	China	*B. minax*	1020	1020	99%	0.0	99%	HM776033
19	*B. oleae*	Adult	Italy	*B. oleae*	1027	1027	100%	0.0	100%	KT369103
20	*B. rubigina*	Adult	China	*B. melastomatos*	1027	1027	100%	0.0	100%	KT881557
21	*B. scutellaris*	Adult	China	*B. diaphora*	905	905	100%	0.0	96%	KT159730
22	*B. scutellata*	Adult	China	*B. scutellata*	1022	1022	100%	0.0	99%	KT159731
23	*B. synnephes*	Adult	China	*B. chorista*	955	955	100%	0.0	98%	JQ671163
24	*B. tau*	Adult	China	*B. tau*	1027	1027	100%	0.0	100%	KP711431

25		*B. thailandica*	Adult	Thailand	*B. dorsalis*	961	961	100%	0.0	98%	HQ260726
26		*B. tryoni*	Adult	Australia	*B. tryoni*	994	994	100%	0.0	99%	GQ255825
27		*B. tsuneonis*	Adult	China	*B. tsuneonis*	959	959	99%	0.0	98%	AB095918
28		*B. umbrosa*	Adult	Thailand	*B. umbrosa*	1000	1000	100%	0.0	99%	KT881558
29		*B. wuzhishana*	Adult	China	*B. dorsalis*	828	828	100%	0.0	94%	AB090272
30		*B. yoshimotoi*	Adult	China	*B. diaphora*	1027	1027	100%	0.0	100%	KT159730
31		*B. zonata*	Adult	Pakistan	*B. zonata*	865	865	86%	0.0	99%	AB192444
32	*Carpomya*	*C. vesuviana*	Adult	China	*C. schineri*	845	845	100%	0.0	94%	EU926793
33	*Ceratitis*	*C. capitata*	Adult	Intercepted	*C. capitata*	1027	1027	100%	0.0	100%	AJ242872
34		*C. cosyra*	Larva	Intercepted	*C. cosyra*	1022	1022	100%	0.0	99%	AY805311
35		*C. rosa*	Larva	Intercepted	*C. rosa*	1005	1005	100%	0.0	99%	EU926795
36	*Dacus*	*D. bivittatus*	Larva	Intercepted	*B. diaphora*	688	688	98%	0.0	89%	KT159730
37		*D. ciliatus*	Larva	Intercepted	*D. ciliatus*	1000	1000	100%	0.0	99%	JQ671195

38		*D. longicornis*	Adult	China	*D. longicornis*	1027	1027	100%	0.0	100%	KX345846
39	*Rhagoletis*	*R. pomonella*	Larva	Intercepted	*R. pomonella*	1022	1022	100%	0.0	99%	KF877736
40		*R.* sp.	Larva	Intercepted	*R. cerasi*	1027	1027	100%	0.0	100%	GQ175823

Figure Legends:

Figure S. Sensitivity of primers TephFdeg/TephR for all tested economically important fruit flies. Template DNA are: Figure S1. *Anastrepha fraterculus*, Figure S2. *Anastrepha oblique*, Figure S3. *Anastrepha sp1*, Figure S4. *Anastrepha sp2*, Figure S5. *Bactrocera albistrigata*, Figure S6. *Bactrocera atrifacies*, Figure S7. *Bactrocera bezziana*, Figure S8. *Bactrocera carambolae*, Figure S9. *Bactrocera caudate*, Figure S10. *Bactrocera cilifera*, Figure S11. *Bactrocera correcta*, Figure S12. *Bactrocera cucurbitae*, Figure S13. *Bactrocera diversa*, Figure S14. *Bactrocera dorsalis*, Figure S15. *Bactrocera hochii*, Figure S16. *Bactrocera kandiensis*, Figure S17. *Bactrocera latifrons*, Figure S18. *Bactrocera minax*, Figure S19. *Bactrocera oleae*, Figure S20. *Bactrocera rubigina*, Figure S21. *Bactrocera scutellaris*, Figure S22. *Bactrocera scutellata*, Figure S23. *Bactrocera synnephes*, Figure S24. *Bactrocera tau*, Figure S25. *Bactrocera thailandica*, Figure S26. *Bactrocera tryoni*, Figure S27. *Bactrocera tsuneonis*, Figure S28. *Bactrocera umbrosa*, Figure S29. *Bactrocera wuzhishana*, Figure S30. *Bactrocera yoshimotoi*, Figure S31. *Bactrocera zonata*, Figure S32. *Carpomya vesuviana*, Figure S33. *Ceratitis capitata*, Figure S34. *Ceratitis cosyra*, Figure S35. *Ceratitis rosa*, Figure S36. *Dacus bivittatus*, Figure S37. *Dacus ciliates*, Figure S38. *Dacus longicornis*, Figure S39. *Rhagoletis pomonella*, Figure S40. *Rhagoletis sp*; DNA serial dilutions are as follows: Lane 1: 100 ng/µl, Lane 2: 10 ng/µl, Lane 3: 1 ng/µl, Lane 4: 0.1 ng/µl, Lane 5: 0.01 ng/µl, Lane 6: 0.001 ng/µl, Lane 7: ddH_2O NTC, Lane M: is a D2000 DNA ladder.

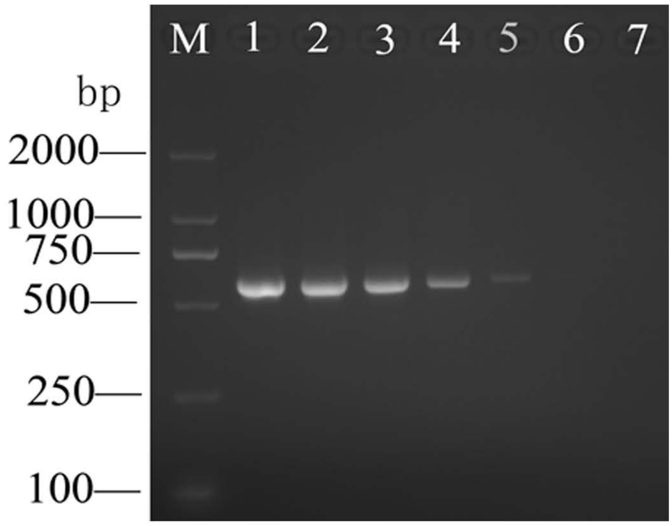

Figure S1. *Anastrepha fraterculus*

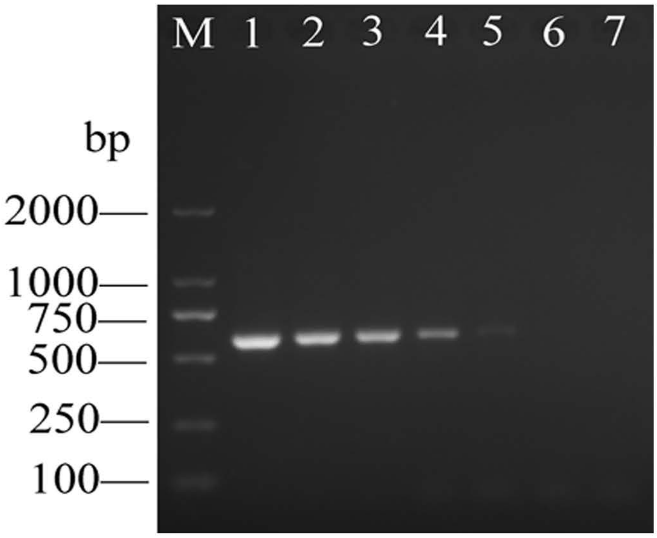

Figure S2. *Anastrepha obliqua*

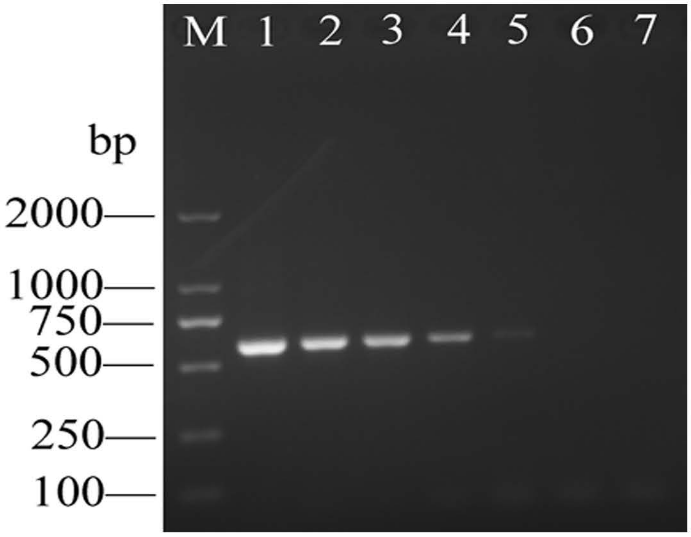

Figure S3. *Anastrepha* sp1

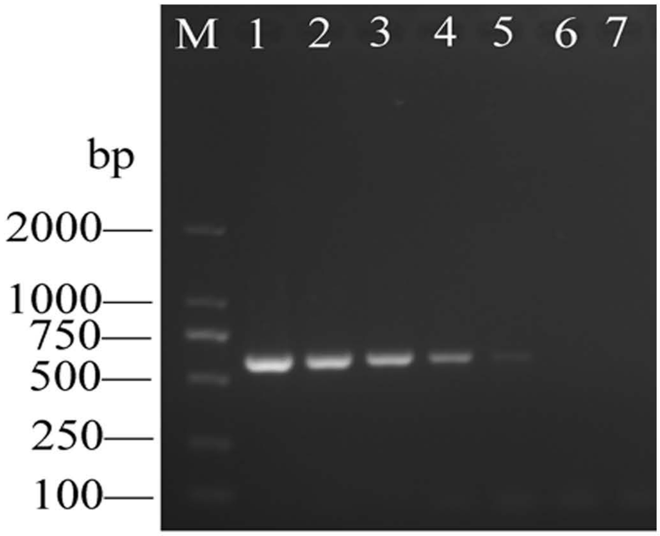

Figure S4. *Anastrepha* sp2

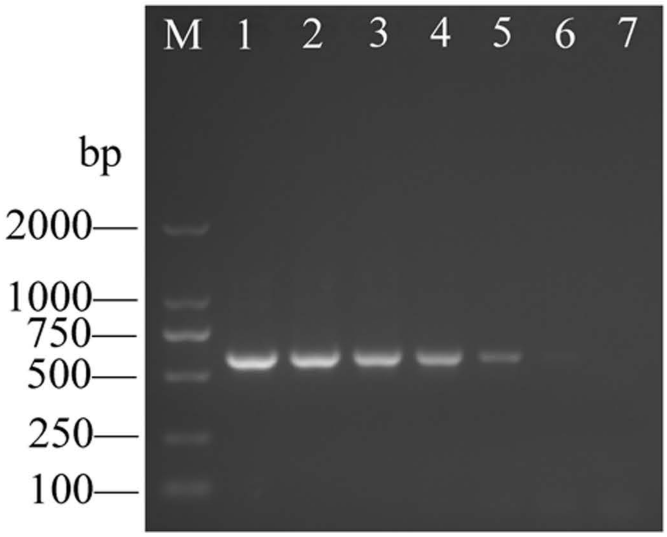

Figure S5. *Bactrocera albistrigata*

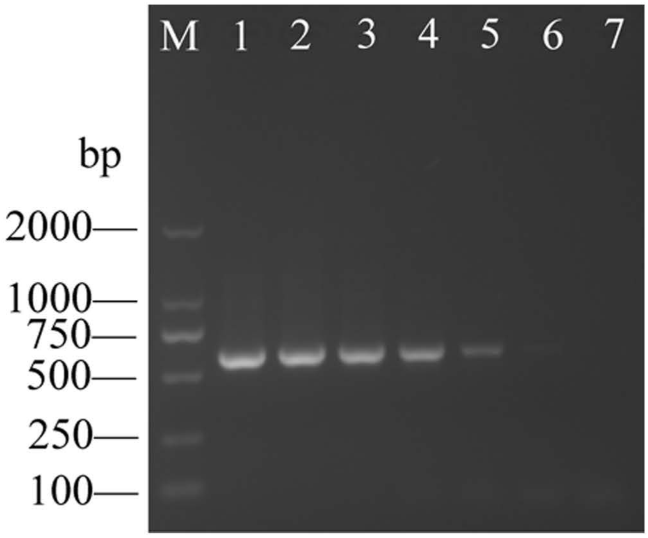

Figure S6. *Bactrocera atrifacies*

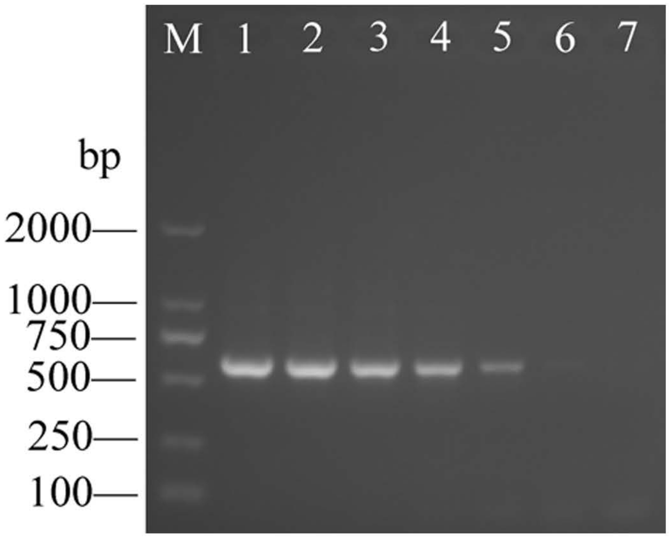

Figure S7. *Bactrocera bezziana*

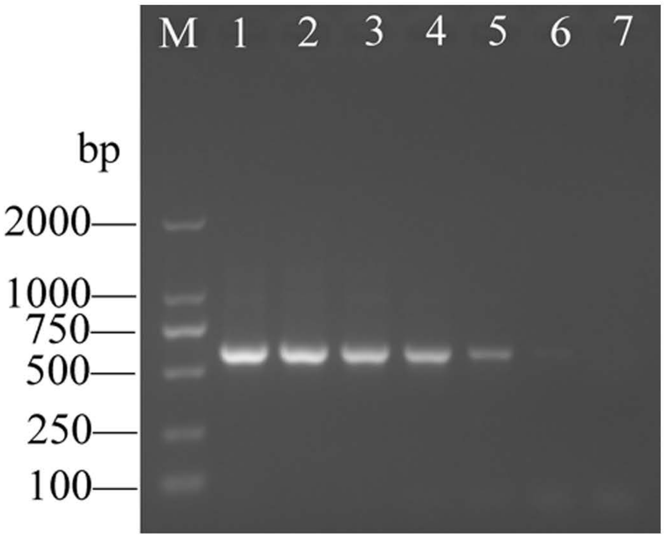

Figure S8. *Bactrocera carambolae*

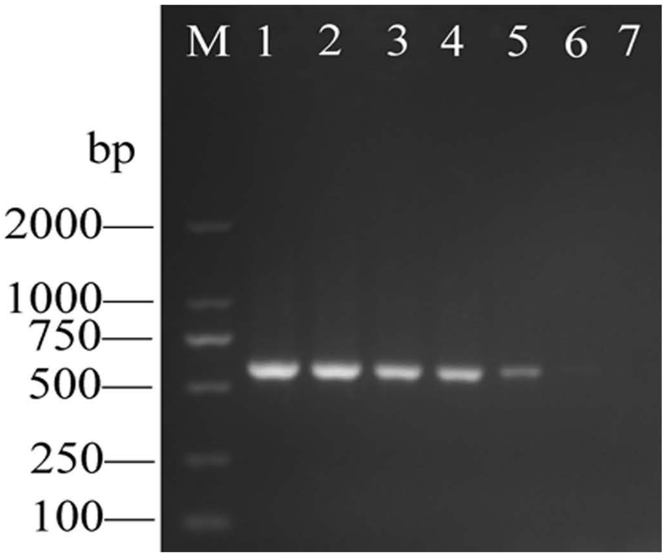

Figure S9. *Bactrocera caudata*

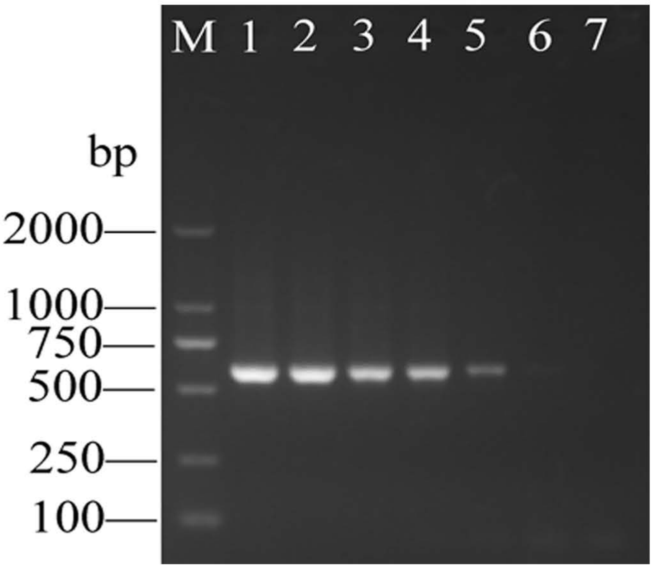

Figure S10. *Bactrocera cilifera*

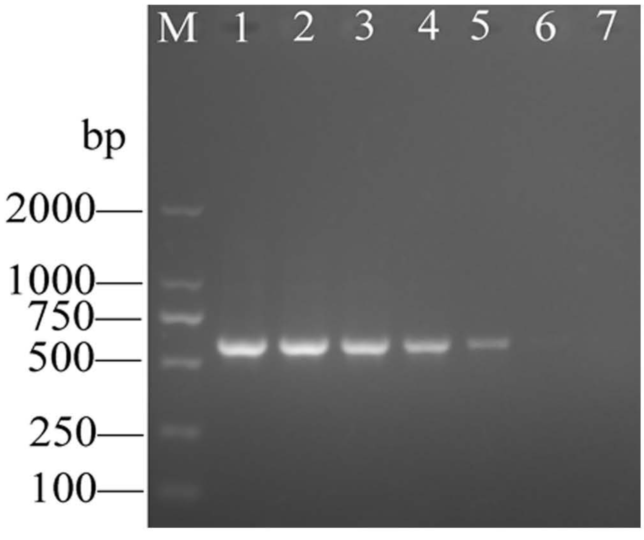

Figure S11. *Bactrocera correcta*

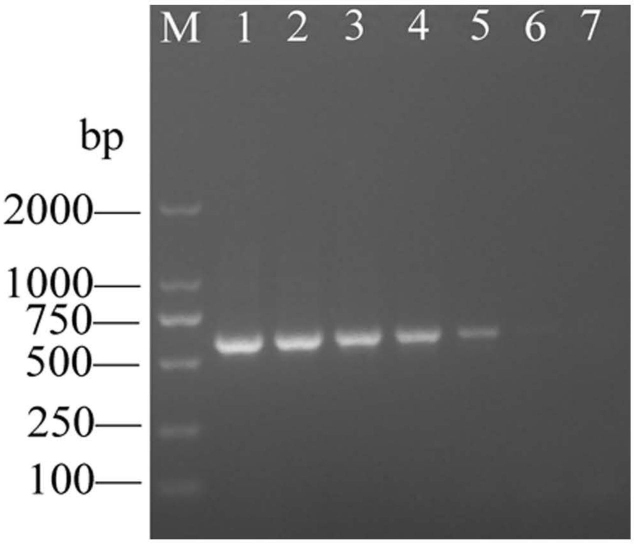

Figure S12. *Bactrocera cucurbitae*

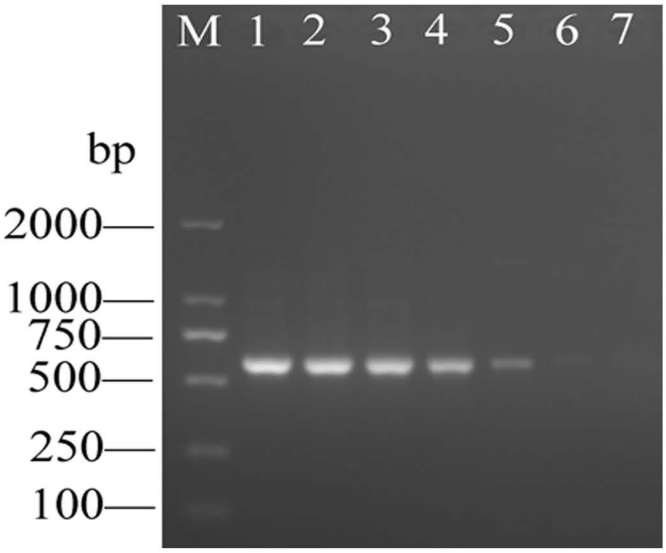

Figure S13. *Bactrocera diversa*

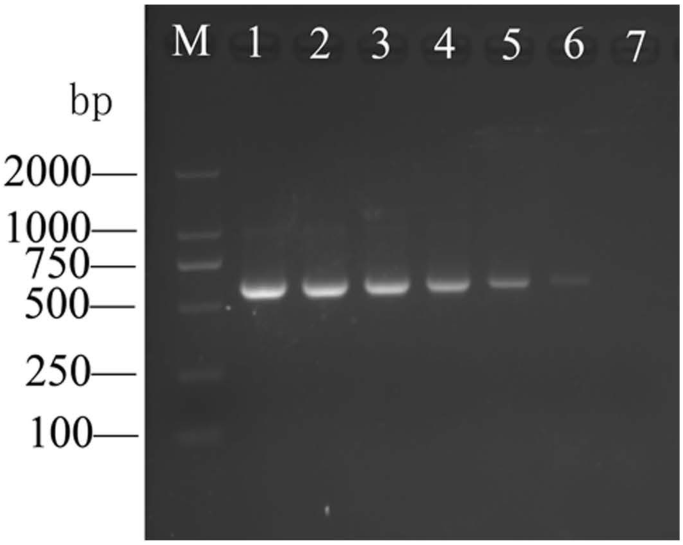

Figure S14. *Bactrocera dorsalis*

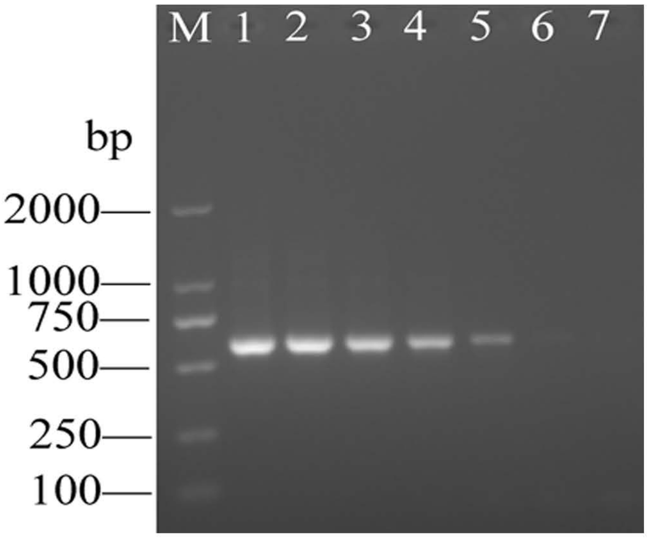

Figure S15. *Bactrocera hochii*

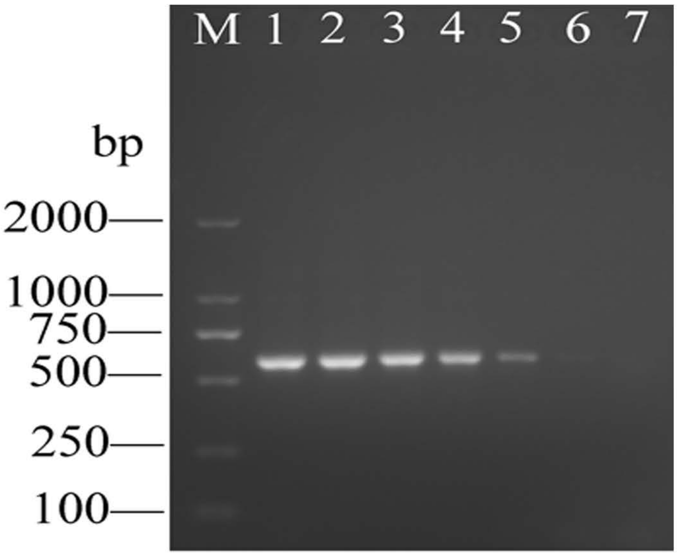

Figure S16. *Bactrocera kandiensis*

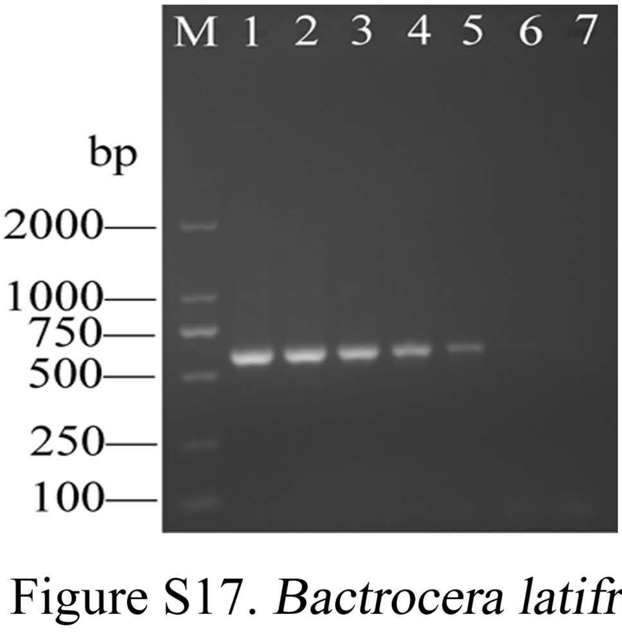

Figure S17. *Bactrocera latifrons*

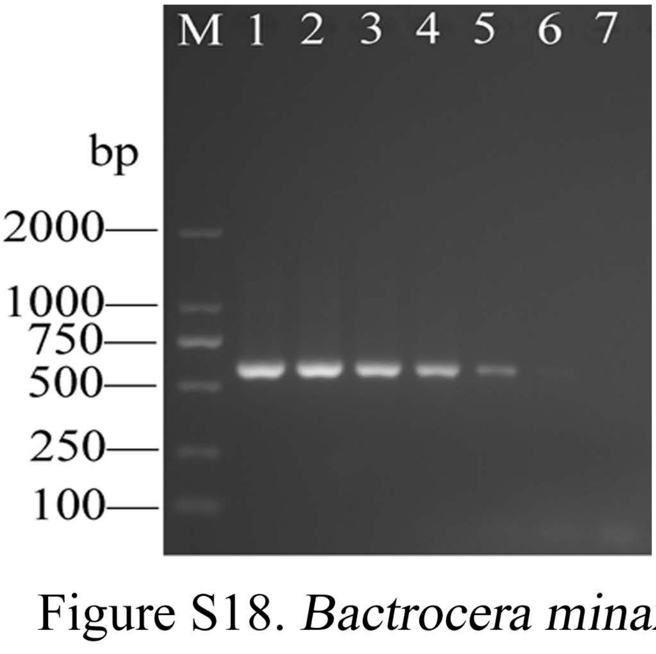

Figure S18. *Bactrocera minax*

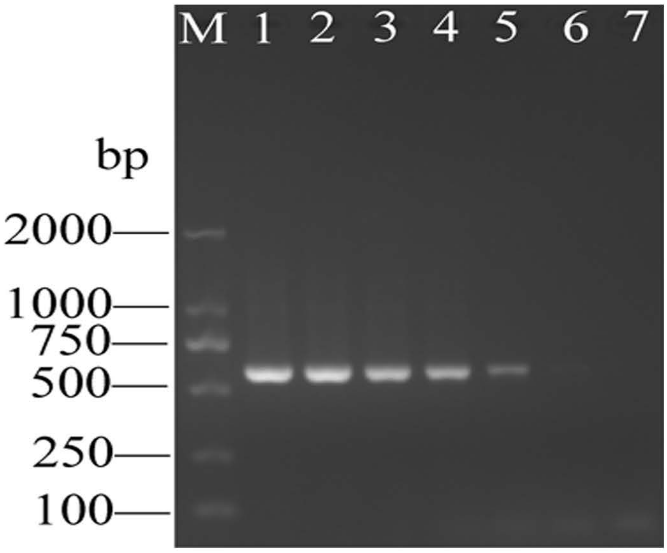

Figure S19. *Bactrocera oleae*

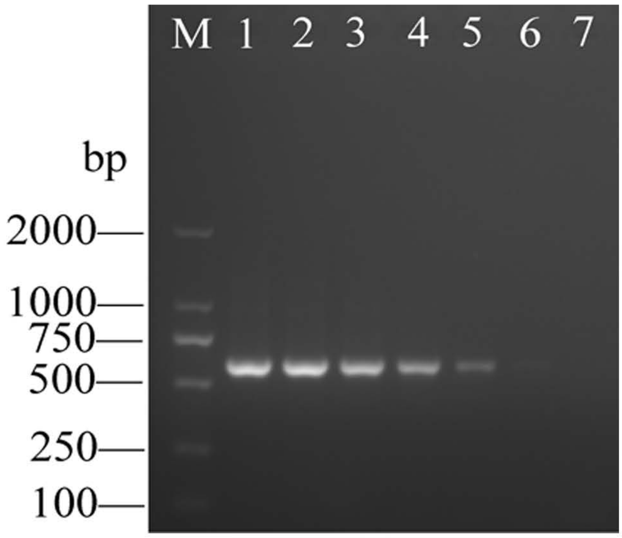

Figure S20. *Bactrocera rubigina*

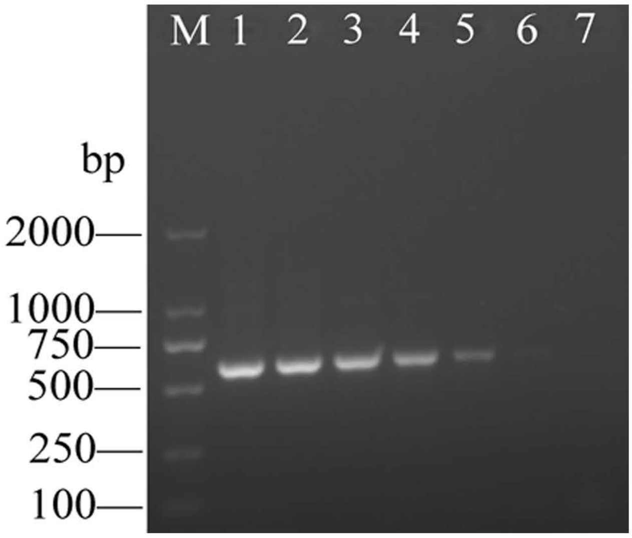

Figure S21. *Bactrocera scutellaris*

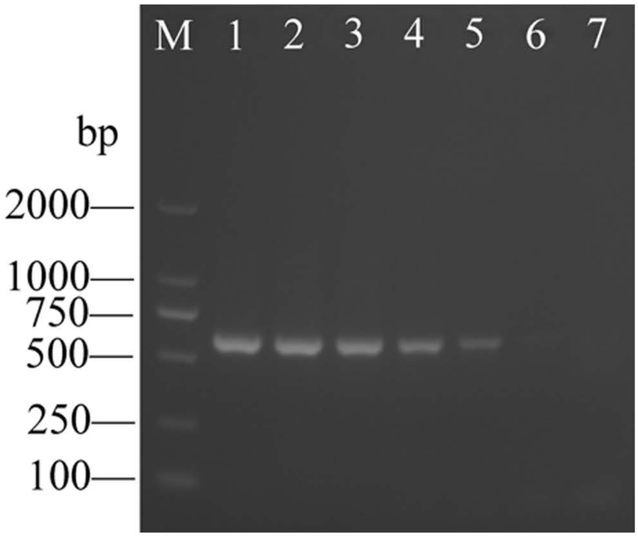

Figure S22. *Bactrocera scutellata*

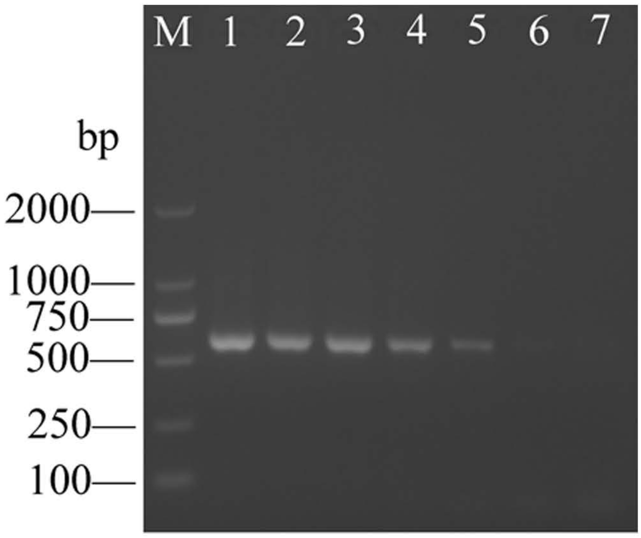

Figure S23. *Bactrocera synnephes*

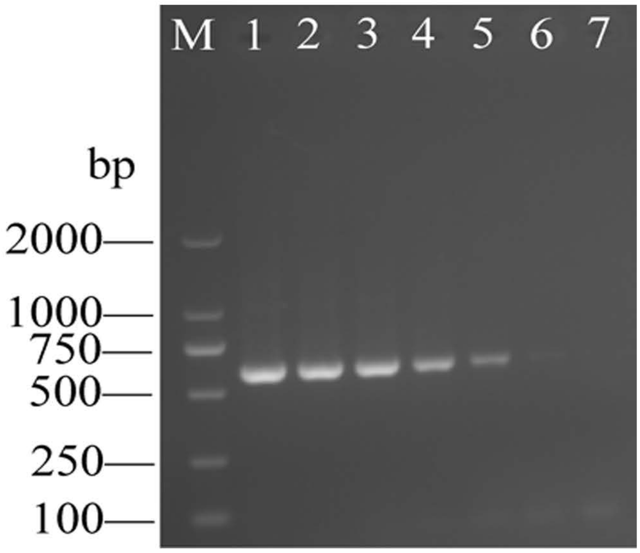

Figure S24. *Bactrocera tau*

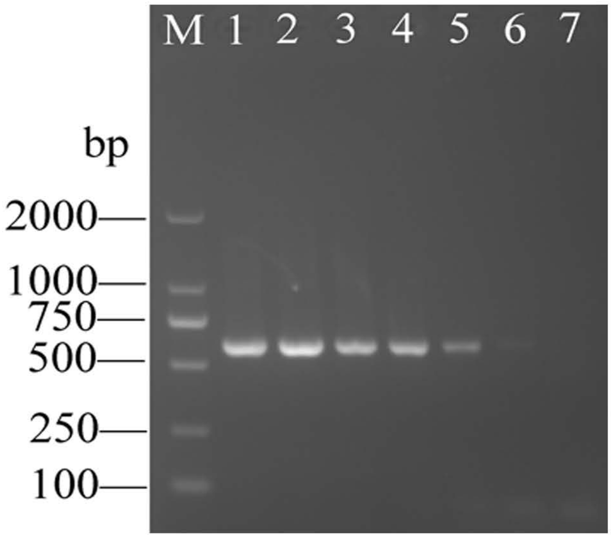

Figure S25. *Bactrocera thailandica*

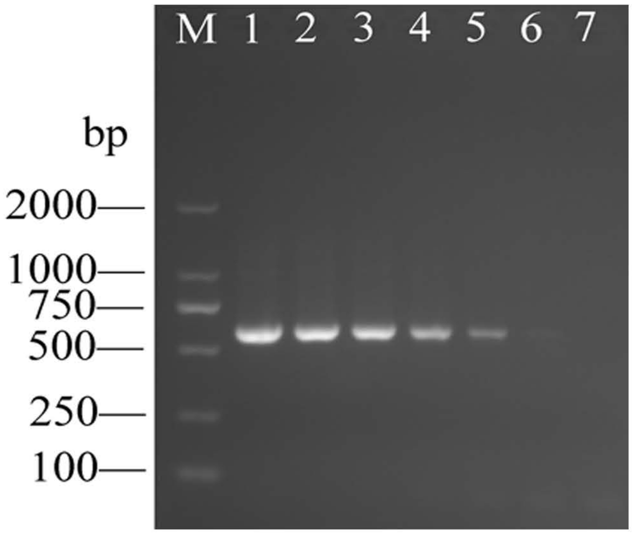

Figure S26. *Bactrocera tryoni*

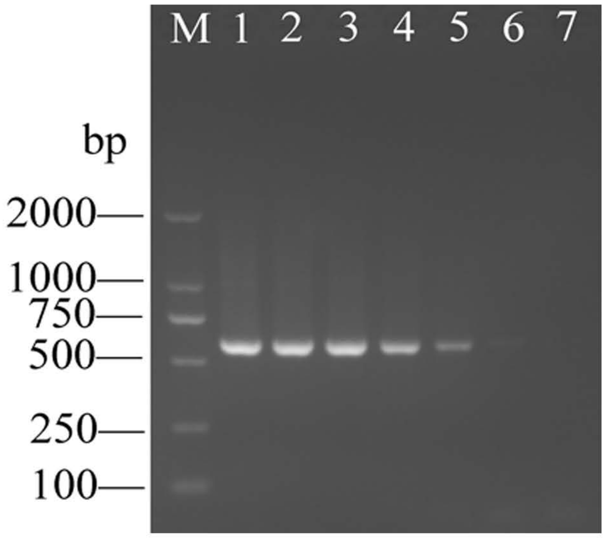

Figure S27. *Bactrocera tsuneonis*

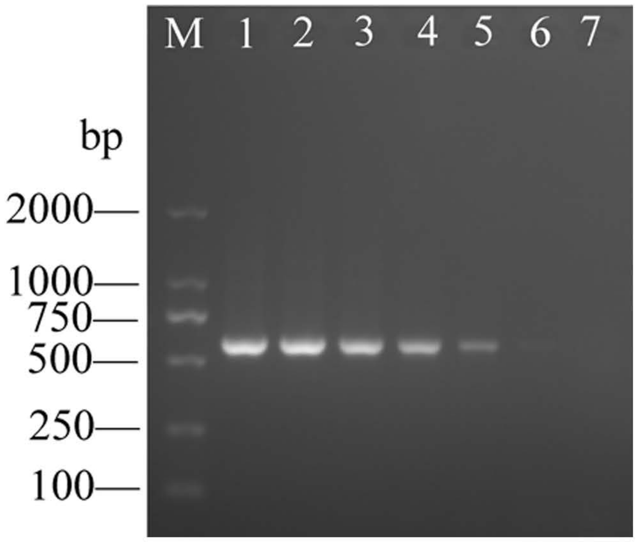

Figure S28. *Bactrocera umbrosa*

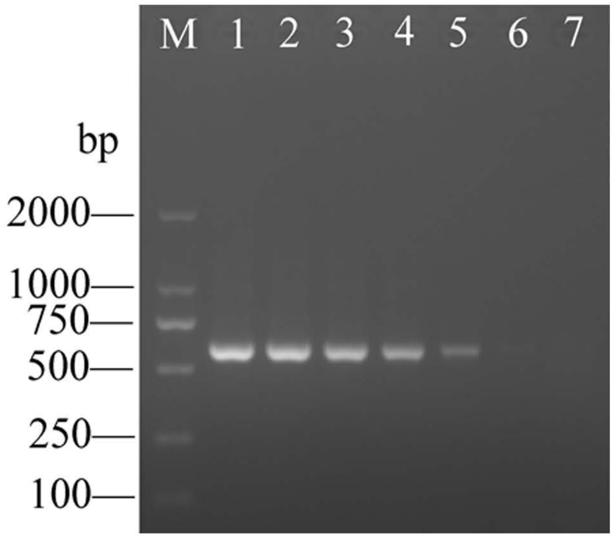

Figure S29. *Bactrocera wuzhishana*

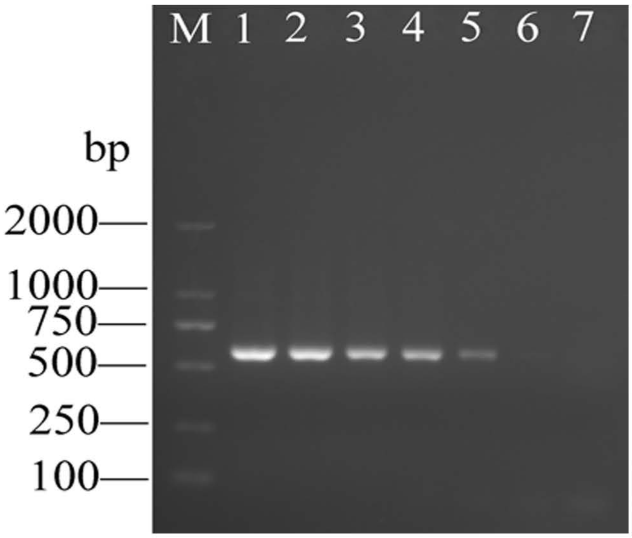

Figure S30. *Bactrocera yoshimotoi*

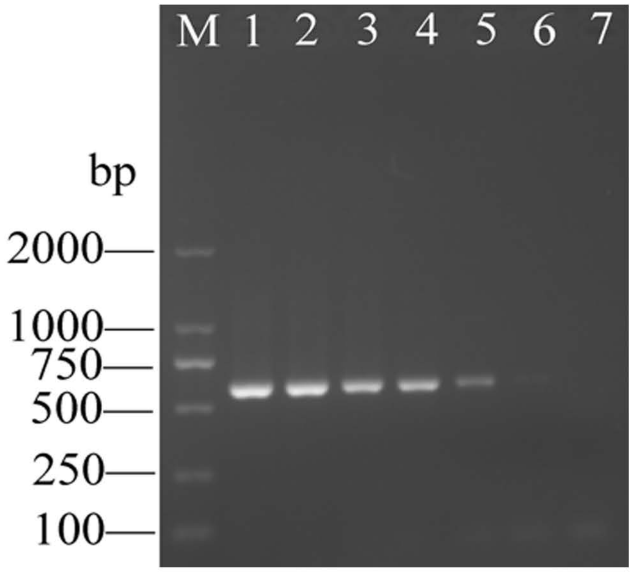

Figure S31. *Bactrocera zonata*

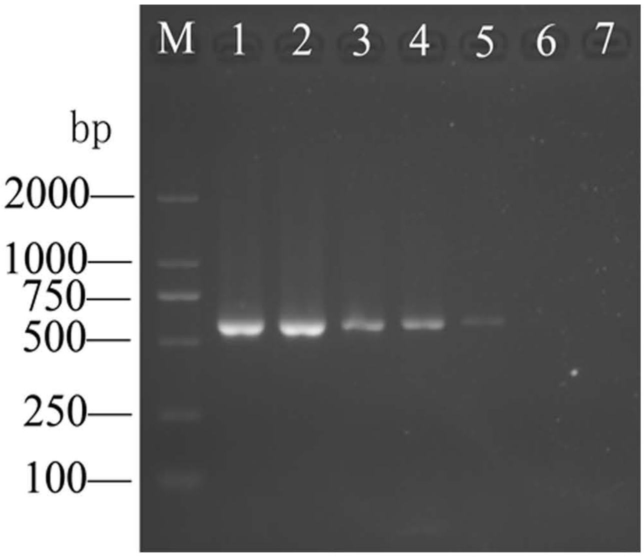

Figure S32. *Carpomya vesuviana*

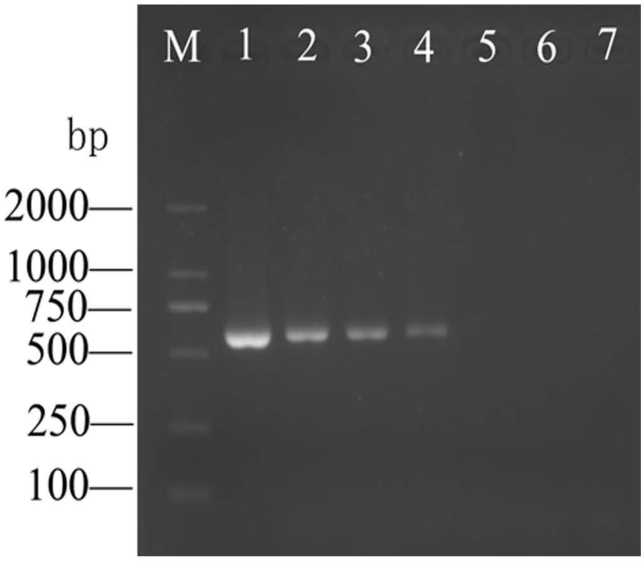

Figure S33. *Ceratitis capitata*

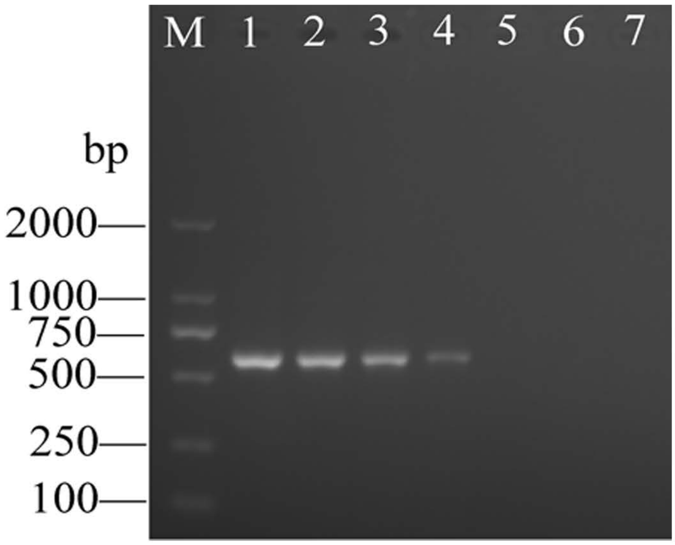

Figure S34. *Ceratitis cosyra*

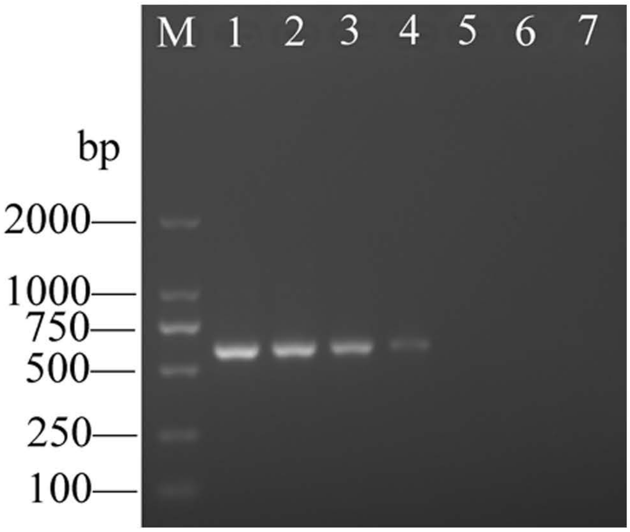

Figure S35. *Ceratitis rosa*

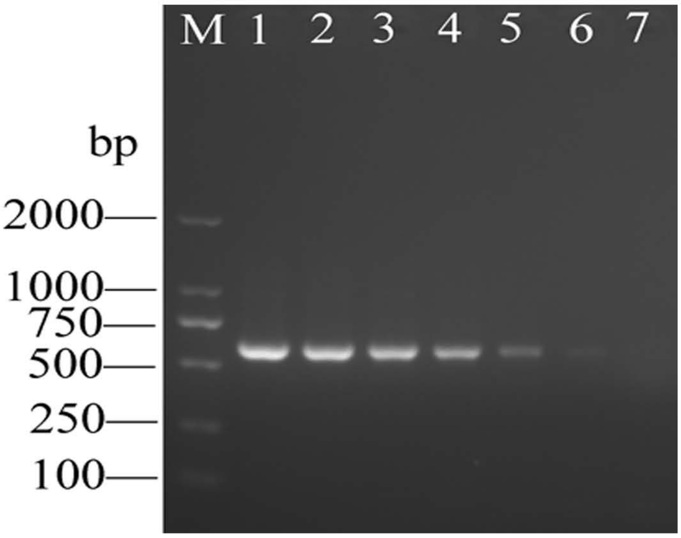

Figure S36. *Dacus bivittatus*

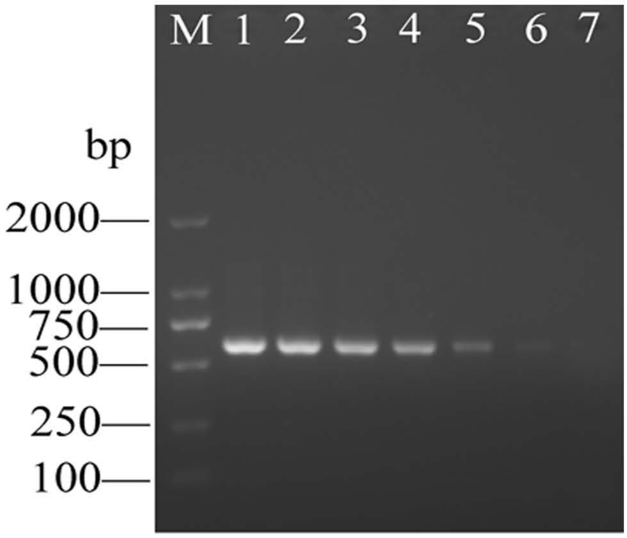

Figure S37. *Dacus ciliatus*

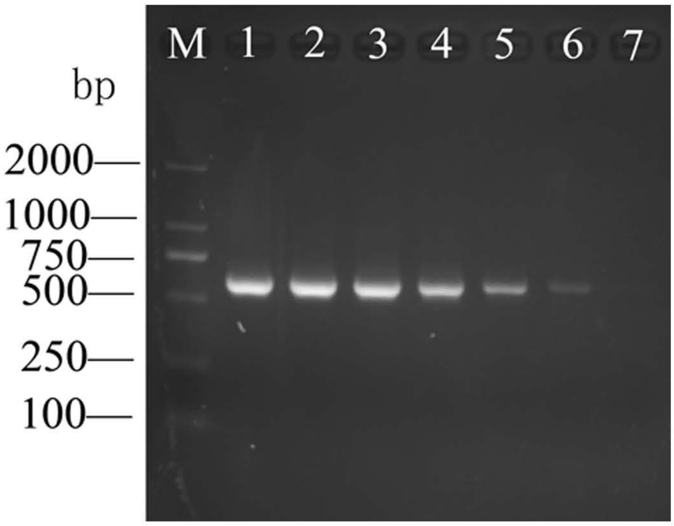

Figure S38. *Dacus longicornis*

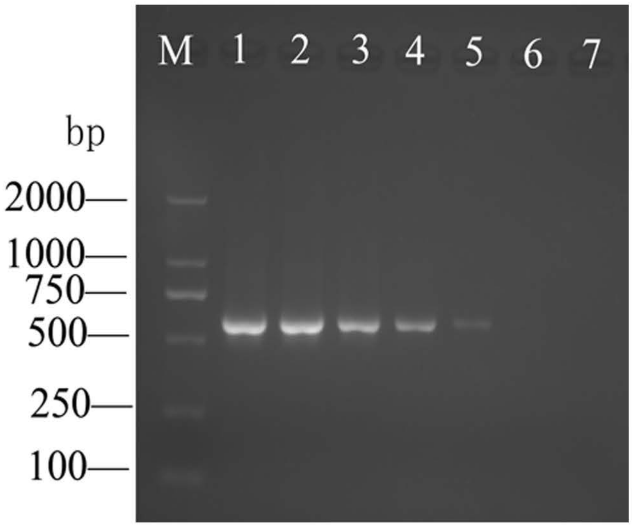

Figure S39. *Rhagoletis pomonella*

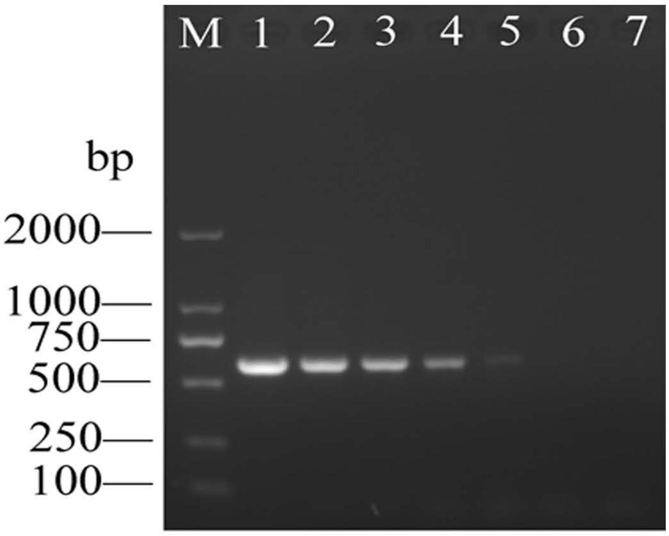

Figure S40. *Rhagoletis sp*
